# Supplementary figures and images for: A new method based on melatonin-mediated seed germination to quickly remove pesticide residues and improve the nutritional quality of contaminated grains
Source: PLoS One. 2024 May 7;19(5):e0303040. doi: 10.1371/journal.pone.0303040 (PMC11075876; doi:10.1371/journal.pone.0303040)

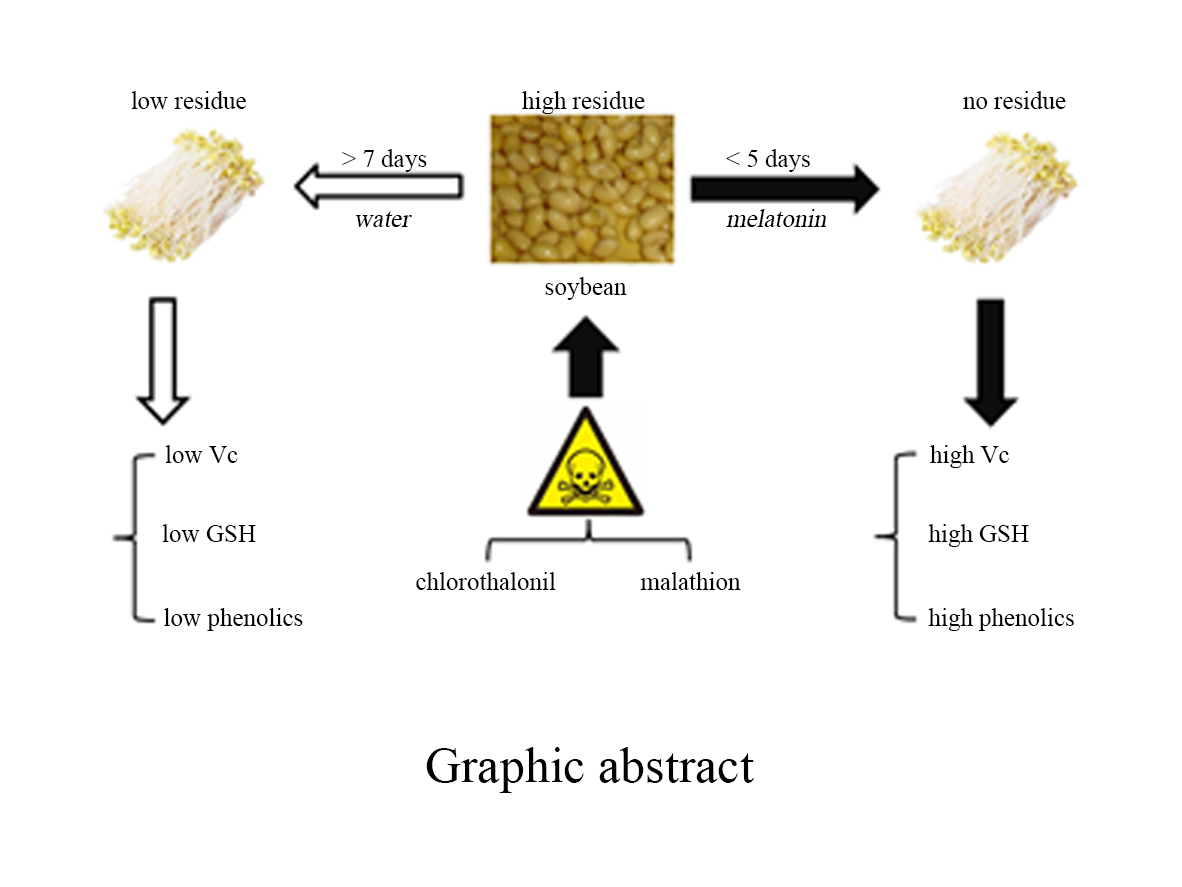

Supplement: S1 File — (ZIP) [file pone.0303040.s001.zip › supporting information file/Graphic abstract.tif]
