# Supplementary material for: A new method based on melatonin-mediated seed germination to quickly remove pesticide residues and improve the nutritional quality of contaminated grains
Source: PLoS One. 2024 May 7;19(5):e0303040. doi: 10.1371/journal.pone.0303040 (PMC11075876; doi:10.1371/journal.pone.0303040)
Supplement: Attachment — Submitted filename: Review comments DBL.docx [file pone.0303040.s002.docx]

Review comments

Dear Sir,

After reviewing the paper entitled “A new method based on melatonin-mediated seed germination for quickly removing pesticide residues and improving nutritional quality for contaminated grains” by Li et al., a new method for removing pesticides from grains contaminated with pesticides has been developed using a combination of melatonin and germination. Not only can it effectively and quickly remove pesticide residues, but it can also increase the accumulation of antioxidants. The experimental design for this work is feasible, the data is reliable, and the necessity of the research work is clearly explained in the introduction. The significance and advantages and disadvantages of the experimental results were also discussed in depth. It is indeed a very interesting and meaningful work. However, there are also some minor problems that need to be improved.

1. Methods. It is recommended to use a flowchart for the experimental design section, which may be more conducive to understanding.
2. It is recommended to include references for the detection of vitamin C.
3. Figure 2 shows the experimental results of melatonin treatment for 24 hours, but the experimental design states that GSH, GR, and GST will be detected after 7 days of treatment. This may be due to the author's mistake or other reasons, which need to be clarified.
4. In the discussion section of L339-341, it is suggested to replace "combined treatment

with exogenous melatonin and pesticides" with "melatonin treatment", which emphasizes the promoting effect of melatonin.

5. At the end of the discussion section, it is suggested to provide suggestions for the shortcomings and improvement measures of this method, which would be better.

Best wishes

Your sincerely

Benlaing Deng, Ph.D
