# Supplementary material for: A new method based on melatonin-mediated seed germination to quickly remove pesticide residues and improve the nutritional quality of contaminated grains
Source: PLoS One. 2024 May 7;19(5):e0303040. doi: 10.1371/journal.pone.0303040 (PMC11075876; doi:10.1371/journal.pone.0303040)
Supplement: Attachment — Submitted filename: Comment response.doc [file pone.0303040.s003.doc]

**Response to comments**

Dear Editor,

I have received the comments for the manuscript entitled “A new method based on melatonin-mediated seed germination to quickly remove pesticide residues and improve the nutritional quality of contaminated grains”(Ms. No. PONE-D-23-43778). I am very grateful for the advice received from the reviewers and the editor. Several major errors have been corrected and they are listed below. I hope that this paper can now be accepted and published in your journal **Plos one**.

Best wishes

You sincerely

Shan Tian, Ph.D.

PONE-D-23-43778

A new method based on melatonin-mediated seed germination for quickly removing pesticide residues and improving nutritional quality for contaminated grains

PLOS ONE

Dear Dr. Tian,

Thank you for submitting your manuscript to PLOS ONE. After careful consideration, we feel that it has merit but does not fully meet PLOS ONE’s publication criteria as it currently stands. Therefore, we invite you to submit a revised version of the manuscript that addresses the points raised during the review process.

Please submit your revised manuscript by May 02 2024 11:59PM. If you will need more time than this to complete your revisions, please reply to this message or contact the journal office at [plosone@plos.org](mailto:plosone@plos.org). When you're ready to submit your revision, log on to https://www.editorialmanager.com/pone/ and select the 'Submissions Needing Revision' folder to locate your manuscript file.

Please include the following items when submitting your revised manuscript:

- A rebuttal letter that responds to each point raised by the academic editor and reviewer(s). You should upload this letter as a separate file labeled 'Response to Reviewers'.
- A marked-up copy of your manuscript that highlights changes made to the original version. You should upload this as a separate file labeled 'Revised Manuscript with Track Changes'.
- An unmarked version of your revised paper without tracked changes. You should upload this as a separate file labeled 'Manuscript'.

If you would like to make changes to your financial disclosure, please include your updated statement in your cover letter. Guidelines for resubmitting your figure files are available below the reviewer comments at the end of this letter.

If applicable, we recommend that you deposit your laboratory protocols in protocols.io to enhance the reproducibility of your results. Protocols.io assigns your protocol its own identifier (DOI) so that it can be cited independently in the future. For instructions see: [https://journals.plos.org/plosone/s/submission-guidelines#loc-laboratory-protocols](https://journals.plos.org/plosone/s/submission-guidelines#loc-laboratory-protocols). Additionally, PLOS ONE offers an option for publishing peer-reviewed Lab Protocol articles, which describe protocols hosted on protocols.io. Read more information on sharing protocols at <https://plos.org/protocols?utm_medium=editorial-email&utm_source=authorletters&utm_campaign=protocols>.

We look forward to receiving your revised manuscript.

Kind regards,

Rahul Kumar Tiwari, PhD
Academic Editor
PLOS ONE

Journal requirements:

1. When submitting your revision, we need you to address these additional requirements.

Please ensure that your manuscript meets PLOS ONE's style requirements, including those for file naming. The PLOS ONE style templates can be found at 
https://journals.plos.org/plosone/s/file?id=wjVg/PLOSOne_formatting_sample_main_body.pdf and 
https://journals.plos.org/plosone/s/file?id=ba62/PLOSOne_formatting_sample_title_authors_affiliations.pdf.

2. We suggest you thoroughly copyedit your manuscript for language usage, spelling, and grammar. If you do not know anyone who can help you do this, you may wish to consider employing a professional scientific editing service.

Whilst you may use any professional scientific editing service of your choice, PLOS has partnered with both American Journal Experts (AJE) and Editage to provide discounted services to PLOS authors. Both organizations have experience helping authors meet PLOS guidelines and can provide language editing, translation, manuscript formatting, and figure formatting to ensure your manuscript meets our submission guidelines. To take advantage of our partnership with AJE, visit the AJE website (http://learn.aje.com/plos/) for a 15% discount off AJE services. To take advantage of our partnership with Editage, visit the Editage website (www.editage.com) and enter referral code PLOSEDIT for a 15% discount off Editage services.  If the PLOS editorial team finds any language issues in text that either AJE or Editage has edited, the service provider will re-edit the text for free.

Upon resubmission, please provide the following:
 
The name of the colleague or the details of the professional service that edited your manuscript

A copy of your manuscript showing your changes by either highlighting them or using track changes (uploaded as a *supporting information* file)

A clean copy of the edited manuscript (uploaded as the new *manuscript* file)”.

3. PLOS requires an ORCID iD for the corresponding author in Editorial Manager on papers submitted after December 6th, 2016. Please ensure that you have an ORCID iD and that it is validated in Editorial Manager. To do this, go to ‘Update my Information’ (in the upper left-hand corner of the main menu), and click on the Fetch/Validate link next to the ORCID field. This will take you to the ORCID site and allow you to create a new iD or authenticate a pre-existing iD in Editorial Manager. Please see the following video for instructions on linking an ORCID iD to your Editorial Manager account: https://www.youtube.com/watch?v=_xcclfuvtxQ.

4. We note that your Data Availability Statement is currently as follows: [All relevant data are within the manuscript and its Supporting Information files.]

Please confirm at this time whether or not your submission contains all raw data required to replicate the results of your study. Authors must share the “minimal data set” for their submission. PLOS defines the minimal data set to consist of the data required to replicate all study findings reported in the article, as well as related metadata and methods (https://journals.plos.org/plosone/s/data-availability#loc-minimal-data-set-definition).

For example, authors should submit the following data:

- The values behind the means, standard deviations and other measures reported;
- The values used to build graphs;
- The points extracted from images for analysis.

Authors do not need to submit their entire data set if only a portion of the data was used in the reported study.

If your submission does not contain these data, please either upload them as Supporting Information files or deposit them to a stable, public repository and provide us with the relevant URLs, DOIs, or accession numbers. For a list of recommended repositories, please see https://journals.plos.org/plosone/s/recommended-repositories.

If there are ethical or legal restrictions on sharing a de-identified data set, please explain them in detail (e.g., data contain potentially sensitive information, data are owned by a third-party organization, etc.) and who has imposed them (e.g., an ethics committee). Please also provide contact information for a data access committee, ethics committee, or other institutional body to which data requests may be sent. If data are owned by a third party, please indicate how others may request data access.

5. Please remove your figures from within your manuscript file, leaving only the individual TIFF/EPS image files, uploaded separately. These will be automatically included in the reviewers’ PDF.

[Note: HTML markup is below. Please do not edit.]

Reviewers' comments:

Reviewer's Responses to Questions

**Comments to the Author**

1. Is the manuscript technically sound, and do the data support the conclusions?

The manuscript must describe a technically sound piece of scientific research with data that supports the conclusions. Experiments must have been conducted rigorously, with appropriate controls, replication, and sample sizes. The conclusions must be drawn appropriately based on the data presented.

Reviewer #1: Yes

Reviewer #2: No

2. Has the statistical analysis been performed appropriately and rigorously?

Reviewer #1: Yes

Reviewer #2: Yes

3. Have the authors made all data underlying the findings in their manuscript fully available?

The [PLOS Data policy](http://www.plosone.org/static/policies.action#sharing) requires authors to make all data underlying the findings described in their manuscript fully available without restriction, with rare exception (please refer to the Data Availability Statement in the manuscript PDF file). The data should be provided as part of the manuscript or its supporting information, or deposited to a public repository. For example, in addition to summary statistics, the data points behind means, medians and variance measures should be available. If there are restrictions on publicly sharing data—e.g. participant privacy or use of data from a third party—those must be specified.

Reviewer #1: Yes

Reviewer #2: Yes

4. Is the manuscript presented in an intelligible fashion and written in standard English?

PLOS ONE does not copyedit accepted manuscripts, so the language in submitted articles must be clear, correct, and unambiguous. Any typographical or grammatical errors should be corrected at revision, so please note any specific errors here.

Reviewer #1: Yes

Reviewer #2: No

5. Review Comments to the Author

Please use the space provided to explain your answers to the questions above. You may also include additional comments for the author, including concerns about dual publication, research ethics, or publication ethics. (Please upload your review as an attachment if it exceeds 20,000 characters)

Reviewer #1: Dear authors,
After reviewing the paper entitled “A new method based on melatonin-mediated seed germination for quickly removing pesticide residues and improving nutritional quality for contaminated grains” by Li et al., a new method for removing pesticides from grains contaminated with pesticides has been developed using a combination of melatonin and germination. Not only can it effectively and quickly remove pesticide residues, but it can also increase the accumulation of antioxidants. The experimental design for this work is feasible, the data is reliable, and the necessity of the research work is clearly explained in the introduction. The significance and advantages and disadvantages of the experimental results were also discussed in depth. It is indeed a very interesting and meaningful work. However, there are also some minor problems that need to be improved.
1. Methods. It is recommended to use a flowchart for the experimental design section, which may be more conducive to understanding.

Response: Thank you for this suggestion. We have added the flowchart.
2. It is recommended to include references for the detection of vitamin C.

Response: Thank you. We have added the references.
3. Figure 2 shows the experimental results of melatonin treatment for 24 hours, but the experimental design states that GSH, GR, and GST will be detected after 7 days of treatment. This may be due to the author's mistake or other reasons, which need to be clarified.

Response: The results measured here indeed reflect the GSH and related enzyme activities after 24 hours of melatonin treatment. Perhaps it was because the experimental methods were not clearly written, leading to misunderstanding. In fact, on the 5th day after melatonin treatment, both pesticide residues disappeared and enzyme activity decreased. Therefore, the higher enzyme activity in Fig. 2 was the result of the data after 24 hours of treatment, not on the 7th day. To avoid misunderstanding, we have revised the experimental section.
4. In the discussion section of L339-341, it is suggested to replace "combined treatment
with exogenous melatonin and pesticides" with "melatonin treatment", which emphasizes the promoting effect of melatonin.

Response: Thank you. Pesticide stimulation can also slightly increase the accumulation of secondary metabolites. However, to emphasize the stimulating effect of melatonin rather than pesticides, we have made revisions according to the reviewer's comments.

5. At the end of the discussion section, it is suggested to provide suggestions for the shortcomings and improvement measures of this method, which would be better.
Response: Thank you for this comment. We have made the requested revisions.

Best wishes
Your sincerely

Reviewer #2: The present manuscript is entitled “A new method based on melatonin-mediated seed germination for quickly removing pesticide residues and improving nutritional quality for contaminated grains”.
The presented manuscript is fascinating. This is extensive research, with a lot of analysis. A special contribution of the manuscript was focused on melatonin-mediated seed germination for quick removal of pesticide residues and improving nutritional quality. I found the research paper appropriate for publication in the Journal, but only after major modifications and clarification from the authors.
Please consider the text at the end of this letter
Keeping in mind the present form a few points which can be incorporated to enhance the quality and visibility of the manuscript

Major Comments
• The abstract portion is weak in a sense as not cover the whole idea of this research.

Response: Thank you, we have improved the abstract.
• The manuscript requires enhancement in its writing. It contains numerous superfluous sentences and grammatical errors that need to be addressed.

Response: Thank you for this comment. The manuscript has been revised by an native English-speaking editor.
• Must write about the specification of the Gas chromatography instrument.

Response: Thank you for this comment. We have added the specification of the Gas chromatography instrument.

• Material and method are incomplete not mentioned about melatonin brand, preparation same applied for pesticides applied.

Response: Thank you for this comment. The relevant text has been improved
• Rather than writing this “ Here, an interesting question arises: why can the germination process promote the degradation of pesticide residues in contaminated soybean seeds?” The author must explain the reason for this.

Response: Thank you for this comment. We made a small correction. Our original intention was to explain how melatonin can accelerate the degradation of pesticides in seeds mediated by germination.
• Conclusion needs improvement in writing.

Response: Thank you for this comment. The conclusion section has been improved.
• Discussion is not more input.

Response: Thank you, the discussion has been improved.
Minor Comments
• Must check the spelling mistakes in the whole manuscript like line no. 26 “germination”.

Response: The manuscript has been checked carefully for spelling mistakes; however, in the example you quote, the word is spelled correctly.

6. PLOS authors have the option to publish the peer review history of their article ([what does this mean?](https://journals.plos.org/plosone/s/editorial-and-peer-review-process#loc-peer-review-history)). If published, this will include your full peer review and any attached files.


If you choose “no”, your identity will remain anonymous but your review may still be made public.


**Do you want your identity to be public for this peer review?** For information about this choice, including consent withdrawal, please see our [Privacy Policy](https://www.plos.org/privacy-policy).

Reviewer #1: No

Reviewer #2: **Yes:** Chirag Maheshwari

In addition, we have made corrections to some minor errors found in the paper. For example, the concentration of malathion should be 1 mM instead of 0.1 mM (which has been corrected); We added a melatonin treatment group to the seed germination rate and shoot length in Figure 2, and changed the time to 1, 3, 5, and 7 days, and adjusted it to a bar chart. Finally, the major changes are shown in RED in the revised manuscript. We would like to express our gratitude to the reviewers and editors for their valuable comments on this article.

Best wishes

Your sincerely

Shan Tian, Ph.D.
